# Operative Surgery

**Published:** 1900-07

**Authors:** 


Operative Surgery. By Joseph D. Bryant, M. D., Professor of the Principles and Practice of Surgery, Operative and Clinical Surgery, University and Bellevue Hospital Medical College, etc. In two volumes. $5 each. New York: D. Appleton & Co. 1900.

This work has been entirely rewritten, rearranged, and reset, and so much enlarged that it has become necessary to divide it into two volumes. Many new illustrations, several in color, have been added, and the work as it now stands takes the highest rank. It is, in fact, unique in its excellence. Vol. I, which is now out, is devoted to the general principles of surgery; antiseptics; the control of hæmorrhage; the treatment of operation wounds; the ligature of arteries; operations on veins, capillaries, the nervous system, tendons, ligaments, fasciæ, muscles, bursæ, and bones; amputations, deformities, and plastic surgery.

This work is sufficiently extensive to meet the demand of the profession generally.

The author is one of the great surgeons of the day, and he has given us a book well matured and eminently practical.

